# *Cinnamomum verum* component 2-methoxycinnamaldehyde: a novel antiproliferative drug inducing cell death through targeting both topoisomerase I and II in human colorectal adenocarcinoma COLO 205 cells

**DOI:** 10.3402/fnr.v60.31607

**Published:** 2016-06-07

**Authors:** Kuen-daw Tsai, Jonathan Cherng, Yi-Heng Liu, Ta-Wei Chen, Ho-Yiu Wong, Shu-mei Yang, Kuo-Shen Chou, Jaw-Ming Cherng

**Affiliations:** 1 Department of Internal Medicine, China Medical University Beigang Hospital, Yunlin, Taiwan ROC; 2 School of Chinese Medicine, College of Chinese Medicine, China Medical University, Taichung, Taiwan ROC; 3 Institute of Molecular Biology, National Chung Cheng University, Chiayi, Taiwan ROC; 4 Faculty of Medicine, Medical University of Lublin, Lublin, Poland; 5 Department of Family Medicine, Saint Mary's Hospital Luodong, Yilan, Taiwan ROC; 6 Department of Internal Medicine; Saint Mary's Hospital Luodong, Yilan, Taiwan ROC

**Keywords:** 2-methoxycinnamaldehyde, antiproliferative, topoisomerase I, topoisomerase II, lysosomal vacuolation, cytotoxicity, xenograft

## Abstract

**Background:**

*Cinnamomum verum* is used to manufacture the spice cinnamon. In addition, the plant has been used as a Chinese herbal medication.

**Methods:**

We investigated the antiproliferative effect of 2-methoxycinnamaldehyde (2-MCA), a constituent of the cortex of the plant, and the molecular biomarkers associated with tumorigenesis in human colorectal adenocarcinoma COLO 205 cells. Specifically, cell viability was evaluated by colorimetric assay; apoptosis was determined by flow cytometry and morphological analysis with bright field, acridine orange, and neutral red stainings, as well as comet assay; topoisomerase I activity was determined by assay based upon DNA relaxation and topoisomerase II by DNA relaxation plus decatentation of kinetoplast DNA; lysosomal vacuolation and volume of acidic compartments (VACs) were determined by neutral red staining.

**Results:**

The results demonstrate that 2-MCA inhibited proliferation and induced apoptosis as implicated by mitochondrial membrane potential (ΔΨ_m_) loss, activation of both caspase-3 and -9, increase of annexin V^+^PI^+^ cells, as well as morphological characteristics of apoptosis. Furthermore, 2-MCA also induced lysosomal vacuolation with elevated VAC, cytotoxicity, and inhibitions of topoisomerase I as well as II activities. Additional study demonstrated the antiproliferative effect of 2-MCA found in a nude mice model.

**Conclusions:**

Our data implicate that the antiproliferative activity of 2-MCA *in vitro* involved downregulation of cell growth markers, both topoisomerase I and II, and upregulation of pro-apoptotic molecules, associated with increased lysosomal vacuolation. *In vivo* 2-MCA reduced the tumor burden that could have significant clinical impact. Indeed, similar effects were found in other tested cell lines, including human hepatocellular carcinoma SK-Hep-1 and Hep 3B, lung adenocarcinoma A549 and squamous cell carcinoma NCI-H520, and T-lymphoblastic MOLT-3 (results not shown). Our data implicate that 2-MCA could be a potential agent for anticancer therapy.

Colorectal cancer is one of the most common malignancies (1). Despite all advances in the modern therapeutic modalities for the disease, it continues to be the fourth most common cause of cancer death after lung, stomach, and liver cancer (2). Furthermore, the overall 5-year survival rate remains very poor (about 10%) for patients at the metastatic stage of the disease (2, 3). Consequently, there is a need for better treatment of the malignancy.

The genus *Cinnamomum* belongs to the Lauraceae family and includes over 200 aromatic evergreen plants distributed mainly in Asia. *Cinnamomum verum* is an evergreen plant in the genus and is native to Sri Lanka. The cortex of the plant is used to manufacture the spice cinnamon. Furthermore, the cortex has been used as a traditional Chinese herbal medication for various conditions, including improvement of complexion; alleviation of inflammation, fever, and cough; induction of perspiration; and management of circulatory disorders (4, 5). In our ongoing study to explore chemopreventive agents from natural resources, 2-methoxycinnamaldehyde (2-MCA), a component of the cortex of this plant, was discovered to have an antiproliferative effect in human colorectal adenocarcinoma COLO 205 cells.

Cancer is a hyperproliferative disorder. Numerous genetic and epigenetic changes are required to transform normal cells into cancer cells. These alterations control various signaling pathways that cooperate to enable cancer cells with a wide range of biological capabilities required for growing, disseminating and finally killing their host (6). Although antiproliferative drugs may act by different mechanisms, apoptosis is the most common as well as preferred mechanism through which many antiproliferative agents kill and *eradicate* cancer cells (7).

Topoisomerases are enzymes that regulate the topological status of DNA and play crucial roles in maintaining genomic integrity (8). The enzymes relax supercoiled DNA through transient, protein-linked cleavages of either one (type I topoisomerase) or both (type II topoisomerase) of the double-stranded DNA strands (9). In addition to apoptosis, topoisomerase is another important target of antiproliferative agents (10–13).

This diversity of mechanisms of tumorigenesis suggests that there are probably various processes that could be critical targets for prevention of tumor. In an attempt to explore the effects as well as underlying mechanisms of 2-MCA in human colorectal adenocarcinoma COLO 205 cells, we performed a series of experiments to delineate the effects of 2-MCA on proliferation and activities of topoisomerase I and II in COLO 205 cells. Our results implicate that 2-MCA inhibited both topoisomerase I and II activities as well as increased lysosomal vacuolation with elevated volume of acidic compartment (VAC) and cytotoxicity. Finally, 2-MCA induced apoptosis, leading to the inhibition of cell growth, both *in vitro* and *in vivo*.

## Materials and methods

### Materials

Fetal bovine serum (FBS) and RPMI-1640 were purchased from GIBCO BRL (Gaithersburg, MD, USA). 2-Methoxycinnamaldehyde (2-MCA) and dimethyl sulfoxide (DMSO) were purchased from Sigma-Aldrich, Inc. (St. Louis, MO, USA).

### Cell culture

Human colorectal adenocarcinoma COLO 205 cells (American Type Culture Collection CCL-222, American Type Culture Collection, Manassas, VA, USA) were cultured in RPMI-1640 medium, supplemented with 10% (v/v) FBS, 10 U/mL penicillin, 10 µg/mL streptomycin, and 0.25 µg/mL amphotericin B at 37°C with 5% CO_2_.

### XTT assay for cell viability

Cells were cultured in 96-well culture plates (1×10^4^ cells/well). After being cultured for 24 h, the cells were incubated with 10, 20, 40, 80, or 160 µM of 2-MCA for 12, 24, or 48 h. The cell viability was evaluated using the Cell Proliferation Kit II (XTT) (Roche Applied Science, Mannheim, Germany) following the manufacturer's instructions. The absorbance was measured by the Tecan infinite M200 spectrophotometer (Tecan, Männedorf, Switzerland) at 492 nm using a reference wavelength at 650 nm.

### LDH cytotoxicity assay

Cells were cultured in 96-well culture plates (1×10^4^ cells/well). After being incubated for 24 h, cells were treated with different concentrations of 2-MCA for 48 h. LDH activity was evaluated using the LDH-Cytotoxicity Colorimetric Assay Kit from BioVision (Milpitas, CA, USA) following the manufacturer's instructions. The absorbance of the samples at 490 nm was determined using the Tecan infinite M200 spectrophotometer (Tecan, Männedorf, Switzerland). Results are expressed as the percentage of change in activity compared with the control.

### Nuclear fragmentation assay

Nuclear fragmentation assay using acridine orange (AO) was achieved to explore the possible mechanism of inhibitory effect of 2-MCA on proliferation in COLO 205 cells. Briefly, the cells were incubated with different 2-MCA concentrations for 48 h and stained with AO (5 µg/mL) at room temperature (14). The cells were then observed using the Nikon ECLIPSE T*i* fluorescence microscope [with C-FL Epi-fl Filter Cube FITC (excitation and emission wavelengths: 465–495 and 515–555 nm, respectively) and C-FL Epi-fl Filter Cube TRITC (excitation and emission wavelengths: 527.5–552.5 and 577.5–632.5 nm, respectively)].

### Comet assay

Comet assay is a gel electrophoresis–based test that has been used to examine DNA injury in individual eukaryotic cells. The test is versatile, sensitive, and relatively simple to achieve. The limit of sensitivity is about 50 strand breaks per diploid cell. The assay was performed according to the methods described by Olive and Banath (15).

### Assay for volume of acidic compartment

Upregulation of the VAC is a general feature of cells that undergo either necrotic or apoptotic cell death. Furthermore, upregulated VAC could be an indication of dying cells (16). To explore the pathogenetic effects of 2-MCA in the cell line, VAC assay for lysosomes was performed as described previously (14). Briefly, 0.5% neutral red stock solution was prepared in 0.9% saline and filtered. Staining solutions were prepared before each experiment by diluting the stock solution (1:10) in RPMI-1640 medium containing 10% FBS without NaHCO_3_. COLO 205 cells had been seeded in 6 cm dishes at the density of 6250/cm^2^ 24 h before 2-MCA was added. After incubation with different concentrations of 2-MCA for another 48 h, the cells were washed twice with phosphate-buffered saline (PBS) and incubated for 4 min with 4 mL staining solution. The cells were then washed twice with PBS, and the neutral red sample was extracted from cells by adding 3 mL acidified alcohol (50% alcohol, 1% acetic acid, and 49% water) per dish. The optical density (OD) at 540 nm of samples was determined by using the Tecan infinite M200 spectrophotometer (Tecan, Männedorf, Switzerland). All assays were performed in triplicate. The OD value of each sample was subtracted from the OD of the dish without cells to yield a net OD. The neutral red uptake readings for each dish were then normalized for total protein.

### Flow cytometric analysis

To investigate the mechanism responsible for inhibition of cell proliferation by 2-MCA in COLO 205 cells, 5×10^5^ cells were plated in 60-mm dishes 24 h before 2-MCA was added. After incubation with different concentrations of 2-MCA for another 48 h, the cells were harvested and analyzed by the Annexin V-FITC Apoptosis Kit from BioVision (Milpitas, CA, USA) following the manufacturer's protocol using the CyFlow SL Flow Cytometer (Cytecs GmbH, Gorlitz, Germany). Twenty-thousand cells were counted for each determination, and data were analyzed using the FloMax Software (Cytecs GmbH, Gorlitz, Germany).

### Mitochondrial membrane potential assay

Mitochondrial membrane potential was quantified using the mitochondrial-specific fluorescent agent JC-1 (Invitrogen, Carlsbad, CA, USA) following the method of Reers et al. (17). JC-1 is monomer when membrane potentials (ΔΨ_m_) are below 120 mV and the dye fluoresces green (540 nm) following excitation with blue light (490 nm). On the contrary, the dye becomes dimer (J-aggregate) at membrane potentials above 180 mV and fluoresces red (590 nm) following excitation with green light (540 nm). COLO 205 cells were incubated with different concentrations of 2-MCA for 48 h, and then harvested. Then the cells (10^6^ cells/mL RPMI-1640 medium containing FBS) were incubated with 25 µM JC-1 at 37°C for 30 min in the dark. Cells were then washed twice with PBS, resuspended in fresh medium, and examined with a fluorescence microscope. For spectrophotometric analysis, cells were cultured in 96-well culture plates (1×10^4^ cells/well). After being incubated for 24 h, cells were treated with different concentrations of 2-MCA for 48 h. Then the cells were incubated with 25 µM JC-1 at 37°C for 30 min in the dark, washed twice with PBS, and analyzed spectrophotometrically. Variations in the ratio of red (590-nm emission) to green (540-nm emission) fluorescence are implicative of the membrane potential changes (18).

### Assay for caspase activity

To further understand the details in 2-MCA-induced apoptosis, the changes in activities of crucial caspases involved in apoptosis were analyzed. The test is based on the measure of the chromophore 7-amino-4-trifluoromethyl coumarin (AFC) after cleavage from the labeled substrate DEVD- and LEHD-AFC by caspase-3 and -9, respectively. The released AFC gives off a yellow–green fluorescence. COLO 205 cells were incubated with different concentrations of 2-MCA for 48 h and caspase-3 and -9 activities were determined using Fluorometric Assay Kit from BioVision (Milpitas, CA, USA) following the manufacturer's instructions. The AFC light emission was evaluated using the Tecan infinite M200 spectrophotometer (Tecan, Männedorf, Switzerland). Results are expressed as the percentage of change in activity compared to the untreated control.

### Assay for topoisomerase I and II activities

Assays for topoisomerase I as well as II activities were performed using the method of Har-Vardi *et al*. (19).

### In vivo tumor xenograft study

Male nude mice (BALB/c Nude; 6 weeks old) were purchased from the National Science Council Animal Center (Taipei, Taiwan, ROC) and maintained in pathogen-free conditions in accordance with the guidelines and regulations for the care and use of laboratory animals of China Medical University. COLO 205 cells (5×10^6^ cells in 200 µL) were injected subcutaneously (SC) into the flanks of the mice. Tumors were allowed to grow until they reached approximate 75 mm^3^, at that time treatment was initiated. Thirty-two mice were randomly divided into four groups. The mice in the 2-MCA treatment group received intratumoral injection of 5, 10, or 20 mg/kg/day of 2-MCA in a 200 µL volume (the solutions were prepared from stock solution of 2-MCA in DMSO and diluted into appropriate concentrations in PBS) daily. The mice in the control group were treated with an equal volume of vehicle. After transplantation, tumor size was monitored at weekly intervals with calipers, and tumor volume was measured by the hemiellipsoid model formula: tumor volume=1*/*2(4π/3)×(*l/*2)×(*w/*2)×*h*, where *l*=length, *w*=width, and *h*=height.

Specimens were analyzed with fluorescent TUNEL assay using the Quick Apoptotic DNA Ladder Detection Kit (Chemicon, Temecuba, CA, USA) following the manufacturer's instructions.

### Statistical analysis

Data are expressed as means±standard error. The determination of statistical significance was evaluated by one-way analysis of variance (ANOVA), then by the Bonferroni *t*-test for multiple comparisons. A *p* value lower than 0.05 was considered statistically significant.

## Results

### Effects of 2-MCA on cell morphological changes

**Fig. 1:**
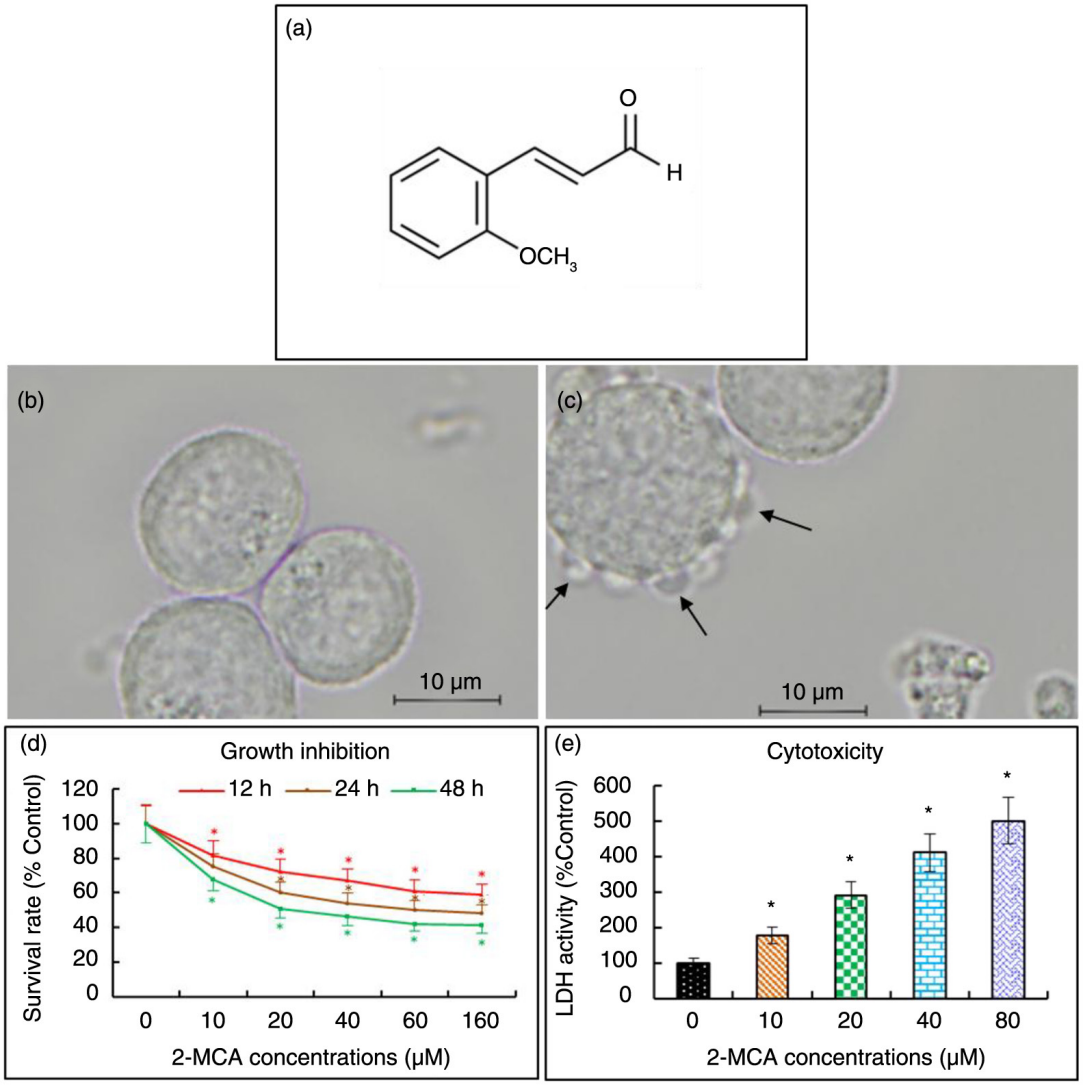
2-MCA's chemical structure and effects on cell morphology, proliferation, and the release of LDH in COLO 205 cells. (a) Chemical structure. (b and c) 2-MCA's effect on cell morphology. Cells were incubated with 0 (b) and 20 (c) µM of 2-MCA for 48 h, respectively. When COLO 205 cells were treated with 20 µM of 2-MCA, blebbing of plasma membrane (arrows), cell shrinkage, as well as cell detachment were observed. (d) 2-MCA's effect of on proliferation. COLO 205 cells were incubated with 2-MCA at the indicated conditions. Cell growth inhibitory activity was measured by the XTT assay. (e) 2-MCA's effect on the release of LDH in COLO 205 cells. The cell culture medium was collected after 48 h of treatment with the indicated concentrations of 2-MCA. Light absorptions were measured using the Tecan infinite M200 spectrophotometer. Data are presented as means±standard error of mean, *n*=3. *Indicates a significant difference (*p*<0.05) from control. 2-MCA, 2-methoxycinnamaldehyde.

When COLO 205 cells were treated with 20 µM of 2-MCA for 48 h, blebbing of plasma membrane was observed. In addition, cell shrinkage as well as cell detachment also occurred (Fig. 1c).

### 2-MCA suppressed COLO 205 cell proliferation

We explored the potential cell growth inhibitory activity of 2-MCA in COLO 205 cells with the XTT assay. As shown in Fig. 1d, 2-MCA suppressed cell growth in COLO 205 cells in a dose- and time-dependent fashion. The *IC*_*50*_ *value* following 48 h of treatment was 22.32 µM.

### 2-MCA induced cytotoxicity in COLO 205 cells

2-MCA was cytotoxic as indicated biochemically by an increase in LDH content in the culture supernatant in a dose-dependent fashion (Fig. 1e).

### 2-MCA induced nuclear fragmentation in COLO 205 cells

**Fig. 2:**
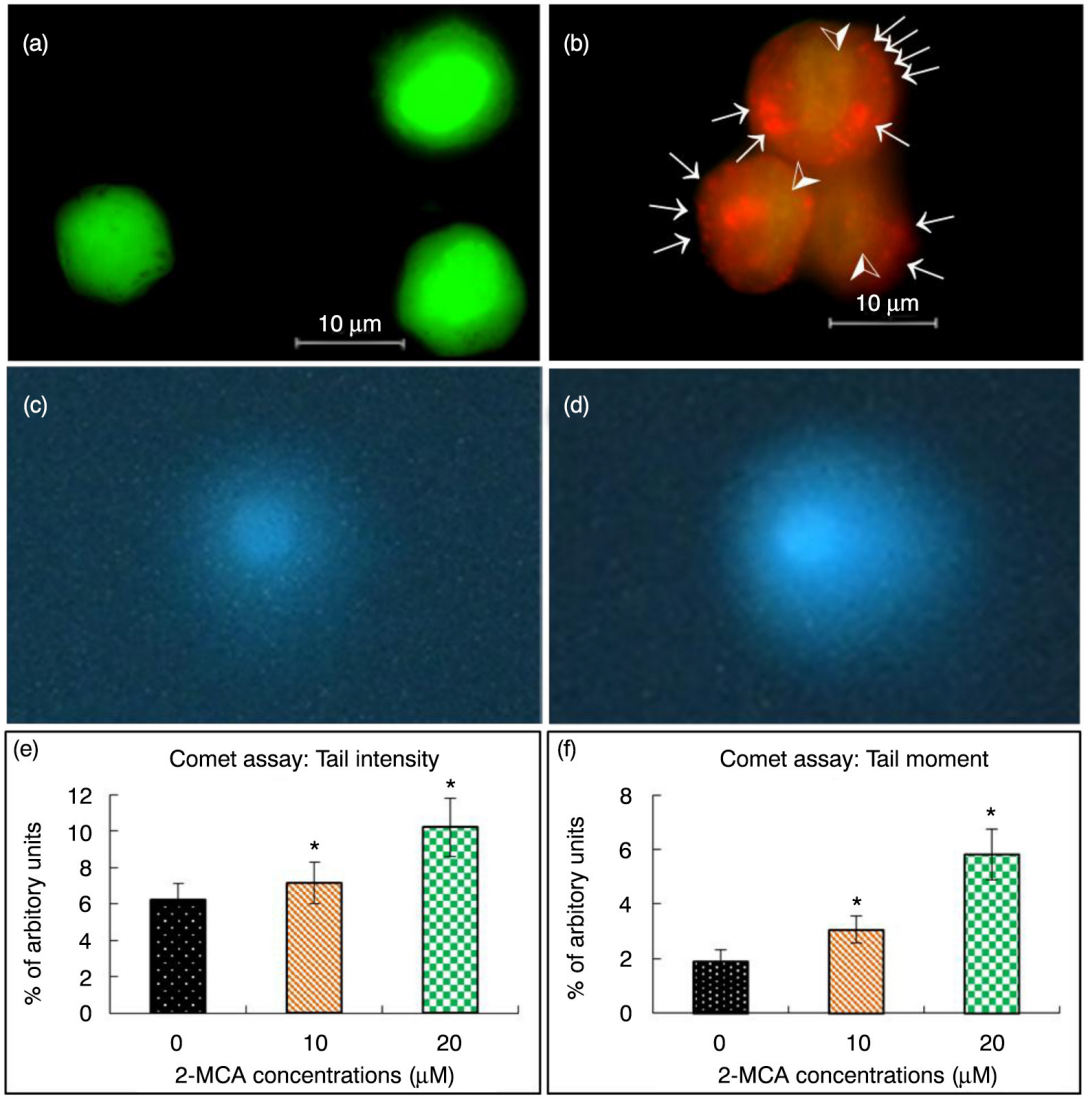
2-MCA induced nuclear fragmentation in COLO 205 cells. (a and b) Acridine orange staining. Cells were treated with 0 (a) and 20 (b) µM of 2-MCA, respectively, for 48 h, then stained with acridine orange. Orange vacuoles in cells show that they were acidic. (a) Representative control group with intact green nucleus suggesting a good cell viability; (b) Representative test group treated with 20 µM 2-MCA in which nuclear fragmentation (arrow heads) and lysosomal vacuolation (arrows) were observed. (c–f) Comet assay. Cells were embedded in agarose and DNA was then unwound in an alkaline solution and subjected to electrophoresis. Cells were then stained with DAPI and analyzed under fluorescence microscope (40× objective). Representative COLO 205 cells treated with 0 (c) and 20 (d) µM of 2-MCA, respectively, for 48 h. Effect of 2-MCA on tail intensity (e) and moment (f). COLO 205 cells were treated with 2-MCA at the indicated conditions for 48 h. Data are expressed as means±standard error of mean, *n*=125. *Indicates a significant difference (*p*<0.05) from control. 2-MCA, 2-methoxycinnamaldehyde.

When COLO 205 cells were incubated with 20 µM of 2-MCA for 48 h, the result of AO staining showed that COLO 205 cells died partially by apoptosis accompanied by nuclear condensation as well as fragmentation. Orange-stained lysosomal vacuoles also appeared. However, no significant nuclear fragmentation was observed in the control group (Fig. 2a).

DNA strand breakage was further explored by comet assay after incubation with different concentrations of 2-MCA. As shown in Fig. 2c–f, 2-MCA treatment resulted in increased tail intensity and moment.

Given that nuclear condensation, fragmentation, blebbing of the plasma membrane, and apoptotic body formation are indicative morphologic features of apoptosis (20), the morphological changes noticed in the study implicate that 2-MCA treatment did lead to apoptosis in COLO 205 cells (Figs. 1c and 2b–d).

### 2-MCA upregulated VACs in COLO 205 cells

**Fig. 3:**
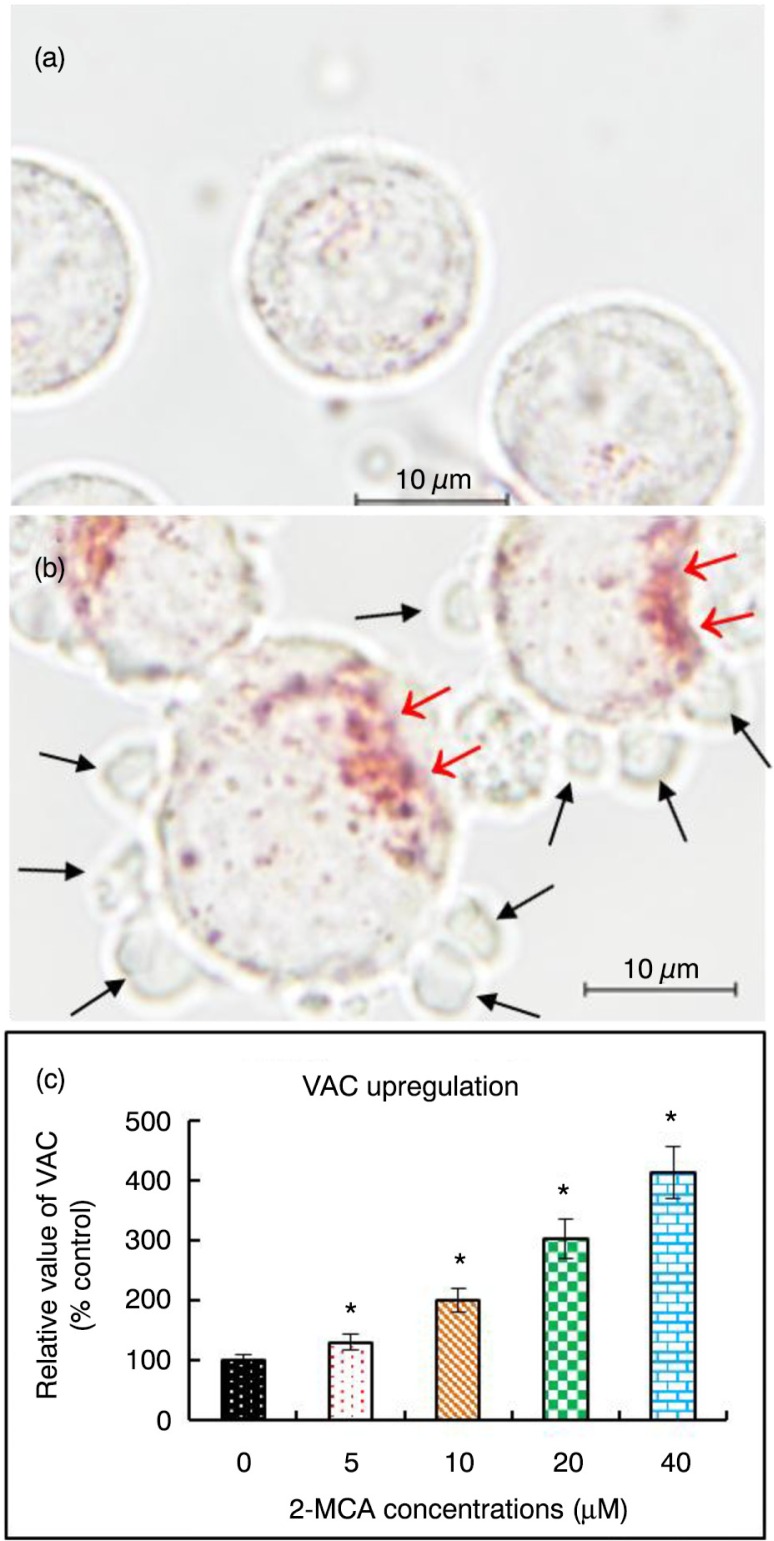
2-MCA upregulated volume of acidic compartment (VAC) in COLO 205 cells. COLO 205 cells were incubated with 0 and 20 µM of 2-MCA, respectively, for 48 h, then stained with neutral red dye. (a) Control group. There were no obvious vacuoles in cells. (b) COLO 205 cells incubated with 20 µM of 2-MCA for 48 h. The acidic red-stained vacuoles (red arrows) and blebbing (black arrows) in cells were noticed. (c) 2-MCA upregulated VAC in a dose-dependent fashion. COLO 205 cells were incubated with the indicated concentrations of 2-MCA for 48 h and data were analyzed using the Tecan infinite M200 spectrophotometer. Data are presented as means±standard error of mean, *n*=3. *Indicates a significant difference (*p*<0.05) from control. 2-MCA, 2-methoxycinnamaldehyde.

Neutral Red dye has been used to stain lysosomes as well as quantify the VAC in cells (14, 21, 22). As shown in Fig. 3a and b, treatment with 2-MCA led to acidic vacuoles in COLO 205 cells, as implicated by positive neutral red staining. Furthermore, as demonstrated in Fig. 3c, the treatment upregulated VAC in a dose-dependent fashion in COLO 205 cells.

### 2-MCA induced apoptosis through the mitochondrial pathway in COLO 205 cells

**Fig. 4:**
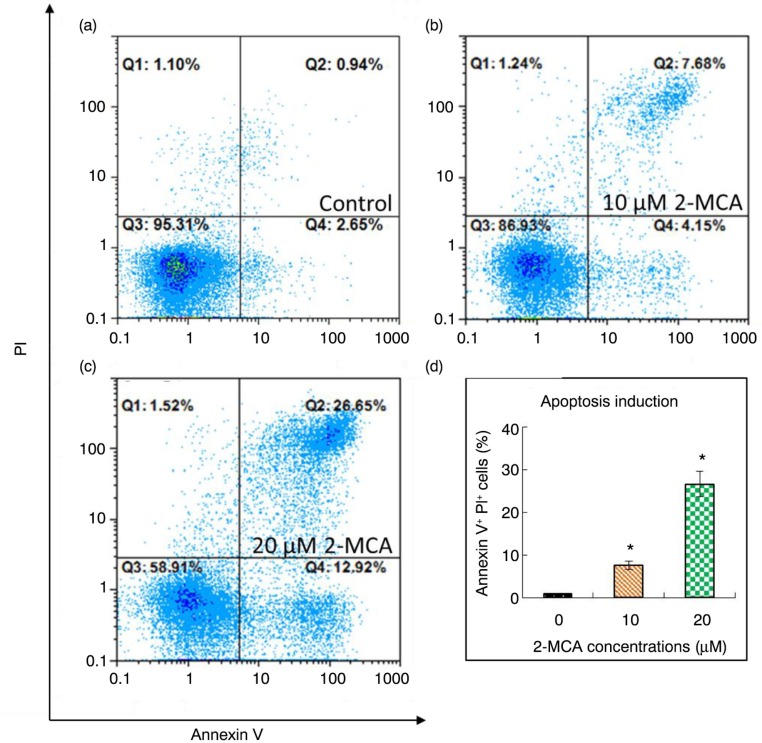
Flow cytometric analysis of 2-MCA-treated COLO 205 cells. (a–c) COLO 205 cells were treated with the indicated concentrations of 2-MCA for 48 h. The induction of cell death was measured at a single-cell level by annexin V and PI staining using CyFlow SL Flow Cytometer. Numbers in each quadrant indicate the percentage of cells among total cells. (d) Quantitative evaluation of annexin V positive/PI positive cells. The data were analyzed using FloMax Software and expressed as means±standard error of mean, *n*=3. *Indicates a significant difference (*p*<0.05) from control. 2-MCA, 2-methoxycinnamaldehyde.

Flow cytometer was used to investigate the mechanism responsible for suppression of cell proliferation by 2-MCA. The Annexin V-FITC and PI staining analysis obtained from COLO 205 cells shows that the treatment with 2-MCA led to increased annexin V positive/PI positive cells. The results demonstrated in Fig. 4d reveals that the percentage of annexin V positive/PI positive cells elevated from untreated cells to cells exposed to 20 µM 2-MCA for 48 h in a dose-dependent fashion.

**Fig. 5:**
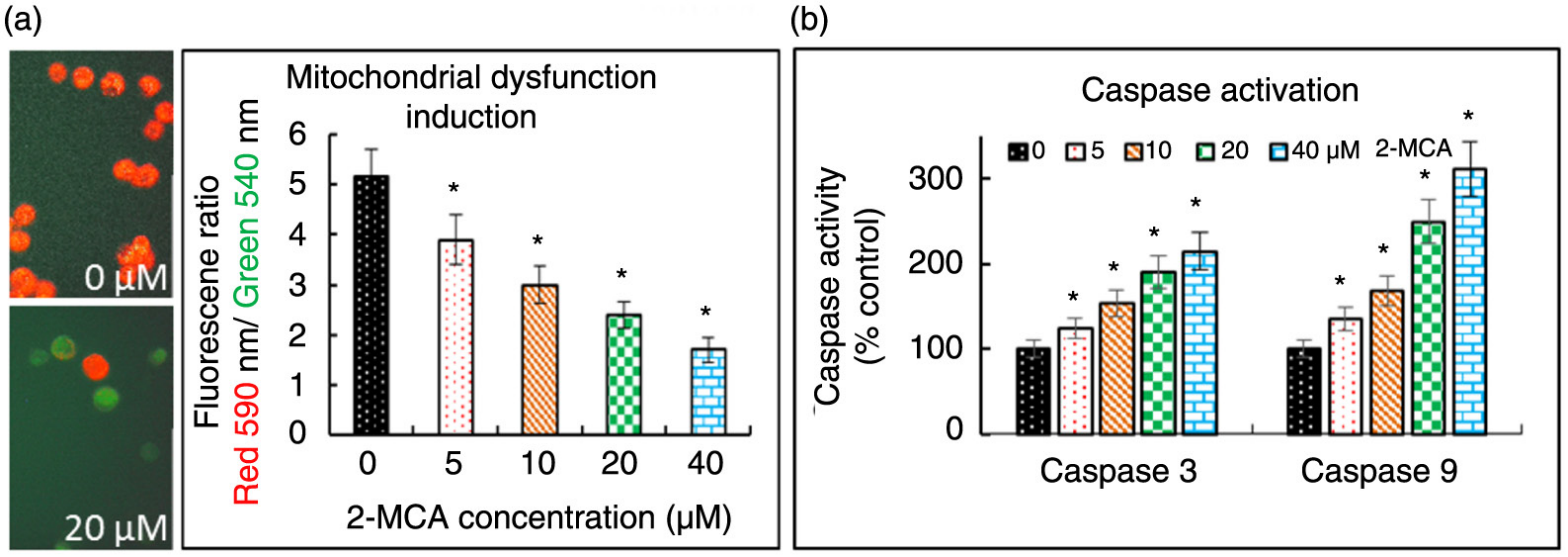
2-MCA induced apoptosis through the mitochondrial pathway in COLO 205 cells. (a) Left, cells were incubated with the indicated concentrations of 2-MCA for 48 h and ΔΨ_m_ was analyzed using JC-1 with a fluorescence microscopy (40× objective). Control cells (upper part) with intact mitochondria fluorescencing red. Most 2-MCA-treated cells (lower part) fluorescencing green, suggesting the loss of ΔΨ_m_. (a) Right, cells were treated with the indicated 2-MCA concentrations for 48 h and ΔΨ_m_ was determined using JC-1 spectrophotometrically. (b) Activations of caspase-3 and -9. The cells were incubated with the indicated 2-MCA concentrations for 48 h and activities of caspases-3 as well as -9 were evaluated using fluorescence-labeled synthetic substrates spectrophotometrically. Data are presented as means±standard error of mean, *n*=3. *Indicates a significant difference (*p*<0.05) from control. 2-MCA, 2-methoxycinnamaldehyde.

We then explored the role of mitochondria in the 2-MCA-induced apoptosis in COLO 205 cells. Because early apoptotic cell death often involves mitochondrial depolarization followed by release of apoptogenic molecules from mitochondria into cytosol, we investigated mitochondrial dysfunction by evaluating mitochondrial membrane potential (ΔΨ_m_) in 2-MCA-treated COLO 205 cells with the mitochondria-specific dye JC-1 using a spectrophotometer. Fig. 5a demonstrates that 2-MCA induced the loss of ΔΨ_m_, as implicated by decrease of ΔΨ_m_ in a dose-dependent fashion.

Caspases are cysteine proteases that play crucial roles in apoptosis. Fig. 5b demonstrates that the activities of caspase-3 as well as -9 increased in a dose-dependent fashion in 2-MCA-treated COLO 205 cells.

### 2-MCA inhibited topoisomerase I activity in COLO 205 cells

**Fig. 6:**
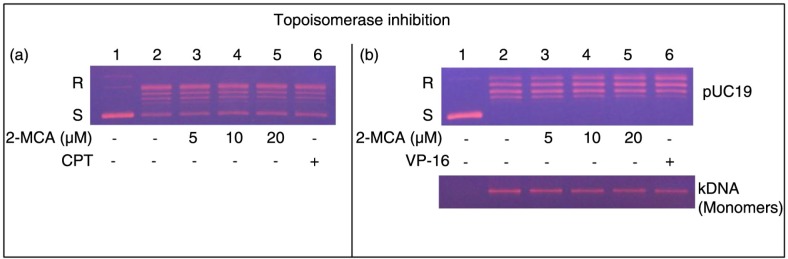
2-MCA suppressed topoisomerase I and II activities in COLO 205 cells. (a) 2-MCA suppressed topoisomerase I activity. COLO 205 cells’ nuclear proteins were added to a specific topoisomerase I reaction mixture with the indicated concentrations of 2-MCA (lanes 3–5), or 60 µM CPT (a specific topoisomerase I inhibitor and used as positive control, lane 6), or the vehicle (1% DMSO, lane 2). Lane 1, pUC19 DNA only. (b) 2-MCA suppressed topoisomerase II activity. DNA relaxation assay (upper part) and decatenation assay (lower part). COLO 205 cells’ nuclear proteins were added to a specific topoisomerase II reaction mixture with the indicated concentrations of 2-MCA (lanes 3–5) or 60 µM VP-16 (a specific topoisomerase II inhibitor and used as positive control, lane 6), or the vehicle (1% DMSO, lane 2). Lane 1, Supercoiled pUC19 DNA (upper part) or kDNA (lower part) only. kDNA is a large network of plasmids. When it is analyzed by gel electrophoresis, it penetrates only slightly into the agarose gel (result not shown). Upon decatenation by topoisomerase II, small circles of monomers of DNA were formed (lower part, lanes 2–6). 2-MCA, 2-methoxycinnamaldehyde; CPT, camptothecin; S and R, supercoiled and the relaxed forms of the pUC 19 plasmid DNA, respectively; VP-16, etoposide.

2-MCA's effect on topoisomerase I activity in COLO 205 cells was achieved using increasing concentration of 2-MCA (Fig. 6a, lanes 3–5) or camptothecin (CPT, a known specific inhibitor of topoisomerase I and used as a positive control, lane 6) (23). Figure 6a demonstrates that the conversion of the supercoiled plasmid pUC 19 to the relaxed form decreased in a dose-dependent fashion in the presence of 2-MCA or CPT (please compare lanes 3–6 with lane 2). These results implicate that 2-MCA inhibited the DNA relaxation activity of topoisomerase I in the COLO 205 cell nuclear proteins.

### 2-MCA inhibited topoisomerase II activity in COLO 205 cells

2-MCA's effect on topoisomerase II activity in COLO 205 cells was explored using increasing concentration of 2-MCA (Fig. 6b, lanes 3–5) or 60 µM etoposide (VP-16, a known inhibitor of topoisomerase II and used as a positive control, lane 6) (24). Figure 6b, upper part, demonstrates that the conversion of the supercoiled plasmid pUC 19 to the relaxed form decreased in a dose-dependent fashion in the presence of 2-MCA or VP-16 (please compare lanes 3–6 with lane 2). These results implicate that 2-MCA inhibited the DNA relaxation activity of topoisomerase II in the COLO 205 cell nuclear proteins. Moreover, this effect was further investigated using the decatenation assay. Decatenation activity involves the release of monomers (mini circular DNA) from the kDNA, a large network of plasmids. The COLO 205 cells’ nuclear proteins contained topoisomerase II, which converted kinetoplast DNA to monomer DNA molecules (Fig. 6b, lower part, please compare lane 2 with lane 1). The conversion of kinetoplast DNA to monomers decreased in a dose-dependent fashion in the presence of 2-MCA (please compare lanes 3–5 with lane 2) or VP-16 (please compare lane 6 with lane 2). These results implicate that 2-MCA inhibited the decatenation activity of topoisomerase II in the COLO 205 cell nuclear proteins.

### 2-MCA inhibited growth of COLO 205 xenograft in nude mice

**Fig. 7:**
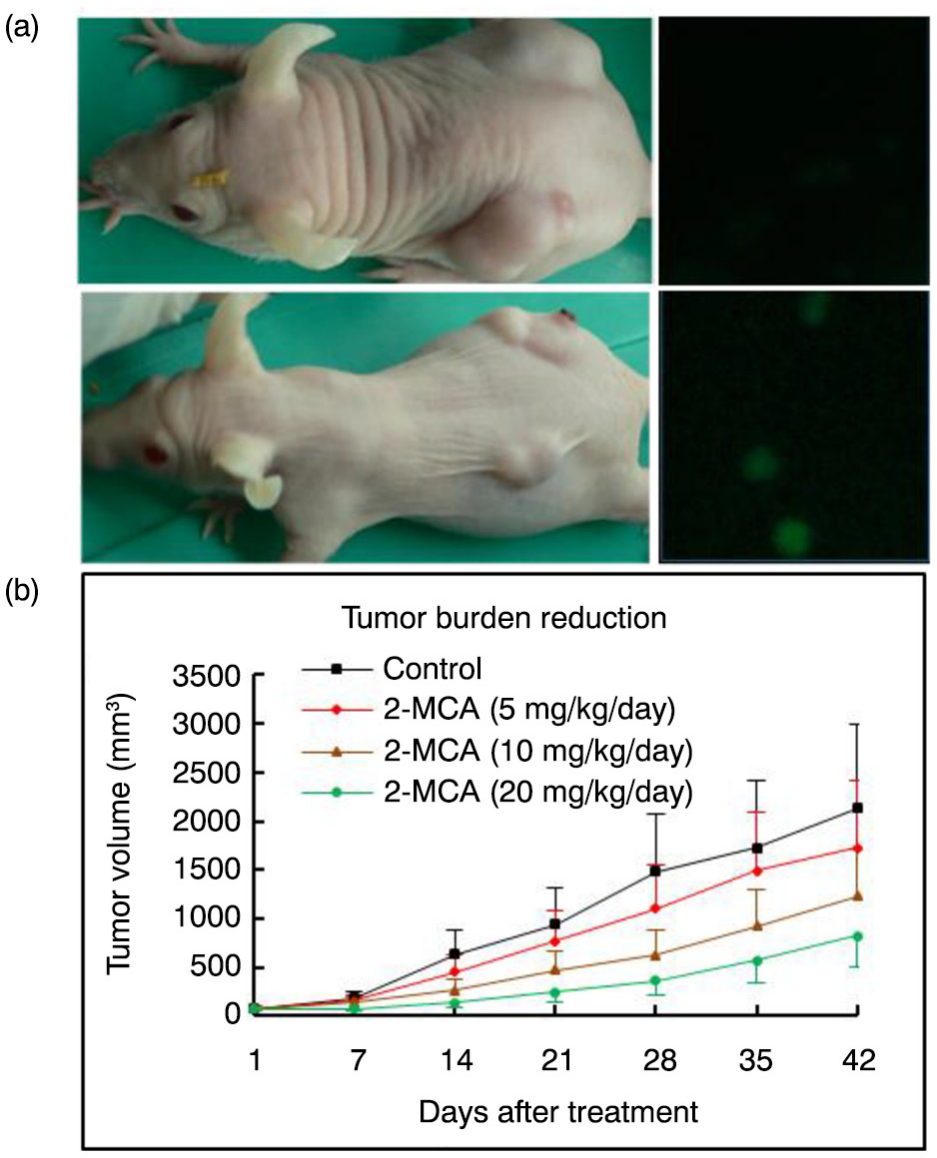
2-MCA inhibited growth and induced apoptosis in COLO 205 xenograft. Animals with pre-established tumors (*n*=8/group) were injected intratumorally with the indicated dosages of 2-MCA. Tumor volumes were monitored by calipers and apoptosis was determined by TUNEL assay. (a) Left, representative tumor-bearing nude mice from the control (upper part) and 20 mg/kg/day of 2-MCA-treated (lower part) groups. (a) Right, 2-MCA induced apoptosis in COLO 205 xenograft by TUNEL assay. Representative TUNEL assay of tumors from the control (upper part) and 20 mg/kg/day of 2-MCA-treated (lower part) groups. (b) Mean of tumor volume recorded at the indicated number of days after initiation of treatment. 2-MCA, 2-methoxycinnamaldehyde.

To explore whether 2-MCA inhibits growth of the COLO 205 xenograft, 5×10^6^ COLO 205 cells in 200 µL of culture medium were used for subcutaneous injection. Fig. 7, left parts, shows that compared with tumors of control mice (upper part), obvious tumor burden reduction was observed in the tumors of the mice treated with 20 mg/kg/day of 2-MCA (lower part). Tumor growth inhibition was observed in all groups with 2-MCA treatment (5, 10, and 20 mg/kg/day of 2-MCA). However, significant growth inhibition was noticed only in mice treated with 10 and 20 mg/kg/day of 2-MCA, where approximately 43.5 and 62.1%reductions in tumor volume were, respectively, observed (Fig. 7b). None of the 2-MCA treatments resulted in any significant reduction in body weight or diet consumption (data not demonstrated) compared with control mice. To investigate the mechanism of the antiproliferative effect of 2-MCA *in vivo*, we harvested the COLO 205 xenograft from vehicle as well as 2-MCA-treated mice and assessed cell death by the TUNEL analysis. Fig. 7a, right parts, shows that compared with tumors of control mice (upper part), increased TUNEL-positive cells that implicate apoptosis were observed in the tumors of the 2-MCA-treated mice (lower part).

## Discussion

Over the past three decades, various approaches have been used for prevention and treatment of cancer at different organ sites, including colon, in various animal models in different laboratories (25–28). Furthermore, in recent years, food and herbal medicines have attracted much attention as potential sources of cancer preventive agents because they are inexpensive, widely available, and applicable for chronic diseases (29, 30).

Traditional Chinese medicine (TMC) demonstrates the enormous accumulation of day-to-day experiences in medical practice by the early Chinese people. The therapeutic usage described in classic books of Chinese materia medica is still informative even in the present day, for example, *Artemisia annua* is efficient against malaria. Artemisinin, an ingredient of the plant, was discovered by Tu Youyou, a Chinese scientist, who was awarded half of the 2015 Nobel Prize in Medicine for her discovery of its effect against *Plasmodium falciparum* malaria. Indeed, various chemical, pharmacological, and clinical studies published today have showed that TCM and Chinese materia medica afford a treasured approach for the research and development of new antiproliferative drugs as illustrated by the examples of irisquinone (from *Iris lactea*) and indirubin (from Danggui Longhui Wan, or Chinese Angelica). In addition, promising data from clinical studies implicate that some herbal medicines could contribute to cancer prevention as illustrated by green tea (28, 31, 32), ginseng (33), *Curcuma longa* (34), garlic (35), and antitumor B (a mixture of Chinese herbs with cancer chemopreventive activity in mouse lung tumor models) (36). Mechanisms of action of tea polyphenols have been extensively investigated and green tea could have a protective effect on lung carcinogenesis (37). Furthermore, the use of chemotaxonomic principle of related species of herbs with reference to the experiences with folk medicine and TCM has furnished an alternative way for the development of new chemopreventive and/or anticancer drugs (38).

Moreover, contemporary experimental and epidemiological studies have persistently revealed that there is an interrelationship between regular consumption of fruits as well as vegetables and prevention of development of lifestyle disorders, such as cancer and cardiovascular disorders (39, 40). Phytochemicals, such as flavonoids and polyphenols which are abundant in vegetables and fruits, seem to have many of the desirable qualities needed for preventing cancer and may have great potential as chemopreventive as well as anticancer agents (41–46). *C. verum* is used to make the spice cinnamon and has been used for treating blood circulation, dyspepsia, and inflammatory disorders, including gastritis (39). 2-MCA, a constituent of the cortex of the tree, could to be such an agent. However, till now very few studies on 2-MCA have been published. The current study thus intended to investigate the antiproliferative activity of 2-MCA as well as elucidate its underlying mechanisms.

In this study, we first explored the 2-MCA's effects on the growth of COLO 205 cells. We found that 2-MCA inhibited the growth of COLO 205 cells in a concentration- and time-dependent fashion. Even though cells could die by non-apoptotic mechanism, apoptosis is the most frequent and preferred mechanism through which various chemotherapeutic agents kill and *eradicate* cancer cells (7). Furthermore, apoptosis has been shown as the major mechanism of tumor cell death induced by several polyphenols (47–50). Our results prove that 2-MCA effectively induced apoptosis as implicated by loss of ΔΨ_m_, upregulation of caspase-3 and -9 (Fig. 5), elevation of annexin V positive/PI positive cells as shown in flow cytometric analysis (Fig. 4), as well as morphological characteristics of apoptosis, such as nuclear condensation, fragmentation, and apoptotic body formation as shown in various stainings and comet assay (Figs. 1–3).

Mitochondria are critical to multicellular life. Apoptosis-inducing agent targeting mitochondria may alter mitochondria through various mechanisms. They could induce the formation of membrane pores resulting in mitochondrial swelling, or elevate permeability of mitochondrial membrane, leading to the release of pro-apoptotic molecules from mitochondrial into cytosol. Then, the released cytochrome *c* interacts with apoptotic protease activating factor-1 (Apaf-1) and deoxyadenosine triphosphate (dATP), which subsequently bind to pro-caspase-9 to generate a protein complex named apoptosome. The apoptosome activates the pro-caspase-9 to active caspase-9. Then, activated caspase-9 activates effector caspase-3, thereby initiating a proteolytic cascade (51–53). Regarding the key events in the induction of apoptosis, the present study shows that 2-MCA induced the collapse of ΔΨ_m_, the increased activities of most upstream protease of the intrinsic apoptotic pathway, caspase-9, as well as the effector caspase-3 implicate the involvement of these pro-apoptotic proteins in 2-MCA-induced apoptosis.

Our results also implicate that 2-MCA induced vacuolation with upregulated VAC. Increase of VAC has been shown to be a common phenomenon found in cells that undergo either necrotic or apoptotic cell death and may be a hallmark of dying cells (16). Since apoptosis is an ordered process, increased VAC could induce the self-digestion during the process of cell death (16).

Topoisomerase I acts by creating a transient single-stranded DNA (ssDNA) break in the DNA double helix, followed by ssDNA crossing or regulated rotation about the break. Topoisomerase I is involved in all DNA processes which involve tracking systems and plays crucial roles in maintaining genomic integrity (8). In addition, elevated levels of topoisomerase I mRNA, protein, as well as catalytic activity have been shown across human tumors (54).

Topoisomerase II acts by generating a transient double-stranded DNA (dsDNA) break, followed by a dsDNA passage event. Topoisomerase II functions in various DNA processes and is needed for decondensation, recombination, separation of daughter chromosomes, condensation, as well as proper chromosome structure (8). The enzyme is upregulated dramatically during cell growth and peaks in the G2/M phase. The resulting transient dsDNA break may lead to fragmentation of the chromosome with chromosomal translocations as well as other DNA abnormalities (9, 55).

In addition to cell cycle regulation, topoisomerase has been shown to be another major target of antiproliferative agents (10–13). The chemotherapeutic drug etoposide kills cancer cells by stabilitating the transient intermediate cleavage complex. The resulting accumulation of cleavage complexes could lead to the formation of permanent DNA strand breaks that fragment the genome leading to the activation of death pathways (56). In addition, apoptosis has been shown to be the most effective death-pathway in tumor cells subsequent to the inhibition of topoisomerase II (57). Our findings document that 2-MCA suppressed topoisomerase I as well as II activities in a dose-dependent fashion (Fig. 6), which, in part, may be a mechanism leading the cells to apoptosis. While most of topoisomerase inhibitors are selectively targeting either topoisomerase I or II (58), our results clearly demonstrate that 2-MCA suppressed topoisomerase I as well as II activities in COLO 205 cells.

It is generally accepted that tumorigenesis is a multi-step process. Investigation on the effects of 2-MCA on topoisomerase I, II, and lysosomal vacuolation in COLO 205 cells could provide novel information on the pathogenetic process of cancer. However, further work is required to elucidate the specific underlying mechanisms of the inhibition, potential mutagenic effects, and other adverse effects for clinical use of 2-MCA as a chemopreventive and/or anticancer drug against human colorectal adenocarcinoma or other malignancies.

Therapy-induced cytotoxicity and other associated adverse effects of antiproliferative agents are major concerns of anticancer therapy. Therefore, the ideal anticancer drug should discriminatorily *kill cancer cells* and not harm the *healthy ones*. Our results demonstrate that none of the 2-MCA treatments induced any significant decrease in body weight or diet consumption compared with control mice. Our results provide convincing evidence of the protective effect of the treatment with 2-MCA against COLO 205 xenograft growth in the present study using nude mice without any observable toxicity; this implicates that 2-MCA has an antiproliferative activity in COLO 205 cells and this agent could potentially serve as a chemopreventive and/or anticancer drug.

Collectively, our data clearly implicate that the antiproliferative activity of 2-MCA in COLO 205 cells *in vitro* involved suppression of cell growth markers, topoisomerase I as well as II, and increase of pro-apoptotic molecules, associated with elevated lysosomal vacuolation. *In vivo*, 2-MCA reduced the tumor burden that could have significant clinical impact.

The present research provides fundamental information on the cancer inhibitory action of 2-MCA in COLO 205 cells that suggests a short-term model for the investigation of potential antiproliferative pharmacological agents against human colorectal adenocarcinoma. Indeed, similar effects were found in other tested cell lines, including human hepatocellular carcinoma SK-Hep-1 and Hep 3B, lung adenocarcinoma A549, squamous cell carcinoma NCI-H520, and T-lymphoblastic MOLT-3 (results not shown). Our results provide a justification for further development of 2-MCA as an effective and safe chemopreventive and/or anticancer drug. Future direction would be to synthesize the derivatives of 2-MCA and examine the protective effects of these agents on key cellular signaling molecules *in vitro*. Then extend the study to examine their effects on growth, progression, and angiogenesis *in vivo*. Finally, use these systems for new drug design and discovery based on parental compound 2-MCA as a lead for safer and more potent chemopreventive and/or anticancer usage.
